# A Flattering Testimonial

**Published:** 1871-07

**Authors:** R. D. Arnold, W. M. Charters, J. G. Thomas, Juriah Harriss, W. Duncan


### A FLATTERING TESTIMONIAL.

An Independent Medical Journal, not devoted to any clique, is a desideratum in our State, for Ethical, Educational and Professional purposes. The undersigned, believing that the GEORGIA MEDICAL COMPANION, published at Atlanta, will fulfil these indications, recommend it to the patronage of all physicians in the State who are desirous of upholding a high standard of Medical Ethics and Medical Education.

R. D. ARNOLD, M. D.
W. M. CHARTERS, M. D.
J. G. THOMAS, M. D.
JURIAH HARRISS, M. D.
W. DUNCAN, M. D.

We return our sincere thanks for the above very flattering endorsement of our Journal, by some of the first professional gentlemen of Savannah. We assure our friends we shall never suffer the COMPANION to lower its flag in the face of an enemy to its principles—which are the principles of the profession.

